# Primary Breast Lymphoma: A Rare Presentation of Diffuse Large B-Cell Lymphoma, Germinal Center B-Cell Subtype (DLBCL-GCB)

**DOI:** 10.7759/cureus.85831

**Published:** 2025-06-12

**Authors:** Dimosthenis Chrysikos, Eirini Livieratou, Stavroula Giannouli, Anna Kontogeorgou, Amir Shihada, Afroditi Nonni, Penelope Korkolopoulou, Theodore Troupis

**Affiliations:** 1 Anatomy, National and Kapodistrian University of Athens School of Medicine, Athens, GRC; 2 Second Department of Internal Medicine, National and Kapodistrian University of Athens School of Medicine, Athens, GRC; 3 First Devision of Pediatrics, "Aghia Sophia" Children's Hospital, National and Kapodistrian University of Athens, Athens, GRC; 4 Pathology, National and Kapodistrian University of Athens School of Medicine, Athens, GRC; 5 First Department of Pathology, National and Kapodistrian University of Athens School of Medicine, Athens, GRC

**Keywords:** diffuse large b-cell lymphoma (dlbcl), germinal center b-cell subtype (gcb), multimodal diagnosis, non-hodgkin lymphoma (nhl), primary breast lymphoma (pbl), r-chop chemotherapy

## Abstract

Primary breast lymphoma (PBL) is a rare type of extranodal lymphoma, accounting for only 0.5% of all breast malignancies. Diffuse large B-cell lymphoma (DLBCL) is the most common histological subtype of PBL, with the germinal center B-cell (GCB) subtype being an exceptionally rare clinical occurrence. This report describes a rare case of primary breast lymphoma of the DLBCL-GCB subtype, presenting as a rapidly enlarging breast mass. A 50-year-old female patient presented with a palpable, painless mass in her left breast. Despite a non-suspicious mammogram (breast imaging-reporting and data system (BI-RADS) 3), the presence of hypoechoic lesions with irregular margins in the ultrasound raised concern (BI-RADS IVc), prompting further imaging. Positron emission tomography-computed tomography (PET-CT) and MRI showed multifocal disease confined to the left breast and ipsilateral axillary lymph nodes, without distant metastasis. Core needle biopsy confirmed the DLBCL-GCB subtype.

The patient was treated with eight cycles of rituximab, cyclophosphamide, doxorubicin, vincristine, and prednisone (R-CHOP) chemotherapy. A mid-treatment PET-CT, performed after four cycles, demonstrated complete regression of hypermetabolic lesions in the left breast. The patient remains in remission on follow-up, suggesting a favorable early outcome. PBL poses diagnostic challenges due to its rarity and clinical overlap with more common breast malignancies. A thorough, multimodal diagnostic approach, combining imaging and histopathology, is essential to ensure accurate diagnosis. Management is primarily based on systemic chemotherapy, often complemented by radiotherapy, with treatment individualized according to disease extent and patient factors.

## Introduction

Distinguishing primary breast lymphoma (PBL) from primary breast carcinoma at presentation is critical, as the two require fundamentally different management. PBL is a rare extranodal lymphoma, accounting for 0.5% of breast malignancies and 1% of all extranodal non-Hodgkin lymphomas. Diffuse large B-cell lymphoma (DLBCL) is the most common subtype of PBL, comprising 56-84% of the cases, although the germinal center B-cell (GCB) subtype is particularly rare.

Unlike primary breast carcinoma, which originates from breast epithelial tissue, PBL arises from lymphoid tissue, making its diagnostic approach especially challenging. Its clinical and radiologic presentation often mimics breast carcinoma, leading to delayed diagnosis or unnecessary surgical interventions. Given the rarity of PBL, especially the DLBC-GBC, and its overlapping clinical features with breast cancer, this case underscores that an accurate diagnosis requires a multimodal diagnostic approach: with imaging, histopathology, and immunohistochemistry. Lastly, early recognition is critical, as the therapeutic strategy for PBL differs significantly from breast cancer, relying primarily on systemic chemoimmunotherapy rather than surgical excision.

## Case presentation

A 50-year-old female presented with a rapidly growing palpable mass in the upper outer quadrant (UOQ) of the left breast. She had a positive family history of breast cancer (two sisters diagnosed in their 40s) but no personal history of malignancy or systemic symptoms. Clinical examination revealed a firm, non-tender mass in the UOQ of the left breast, with no signs of weight loss, night sweats, nipple retraction, or skin changes.

**Figure 1:**
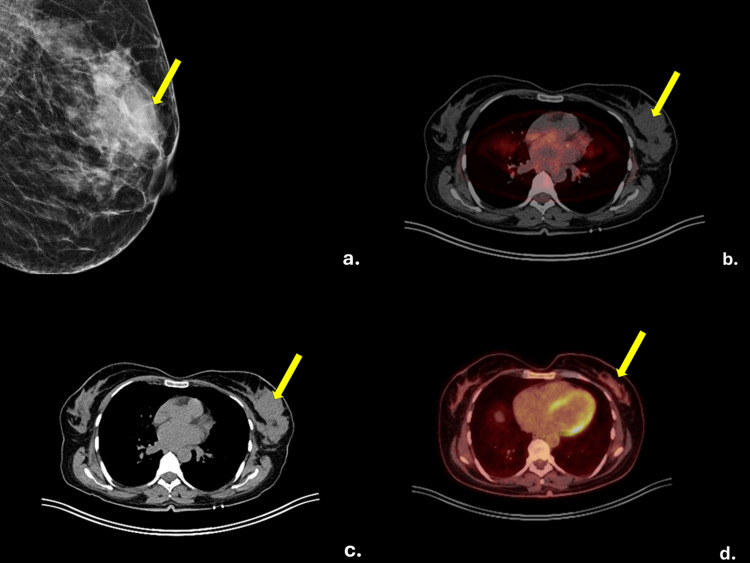
Diagnostic images a. Mammography showing dense breast tissue ACR-type C b. Axial fused positron emission tomography–computed tomography (PET-CT) (pre-treatment) demonstrating intense F-18 fluorodeoxyglucose (FDG) uptake in the left breast mass and ipsilateral axillary lymph nodes, consistent with metabolically active malignancy. c. An axial CT image (pre-treatment) showing a dense soft tissue mass in the UOQ of the left breast, with associated left axillary lymphadenopathy. d. Axial fused PET-CT (midtreatment-4 cycles RCHOP) demonstrating complete metabolic response with no residual FDG uptake in the left breast or axilla. ACR: American College of Rheumatology, RCHOP: Rituximab, Cyclosphamide, Hydroxydaunorubicin, Vincristine (Oncovin), Prednisone.

Breast ultrasound revealed the presence of hypoechoic lesions with irregular margins, the largest of which measured 5.98 × 2.81 cm, in the UOQ of the left breast (breast imaging-reporting and data system (BI-RADS) 4c), without suspicious axillary lymphadenopathy. Mammography, as seen in Figure 1a, showed dense breast tissue (American College of Rheumatology (ACR) type C) without suspicious microcalcifications or architectural distortion (BI-RADS 3-probably benign), but the discordant ultrasound findings prompted further investigation. Thoracic CT, as seen in Figure 1c, identified ipsilateral axillary lymphadenopathy (largest node 35 × 20 mm) and two small non-FDG-avid pulmonary nodules, favoring benign etiology. Positron emission tomography-computed tomography (PET-CT), as seen in Figure 1b, demonstrated disease confined to the left breast and ipsilateral axilla, with no evidence of distant metastasis.

**Figure 2:**
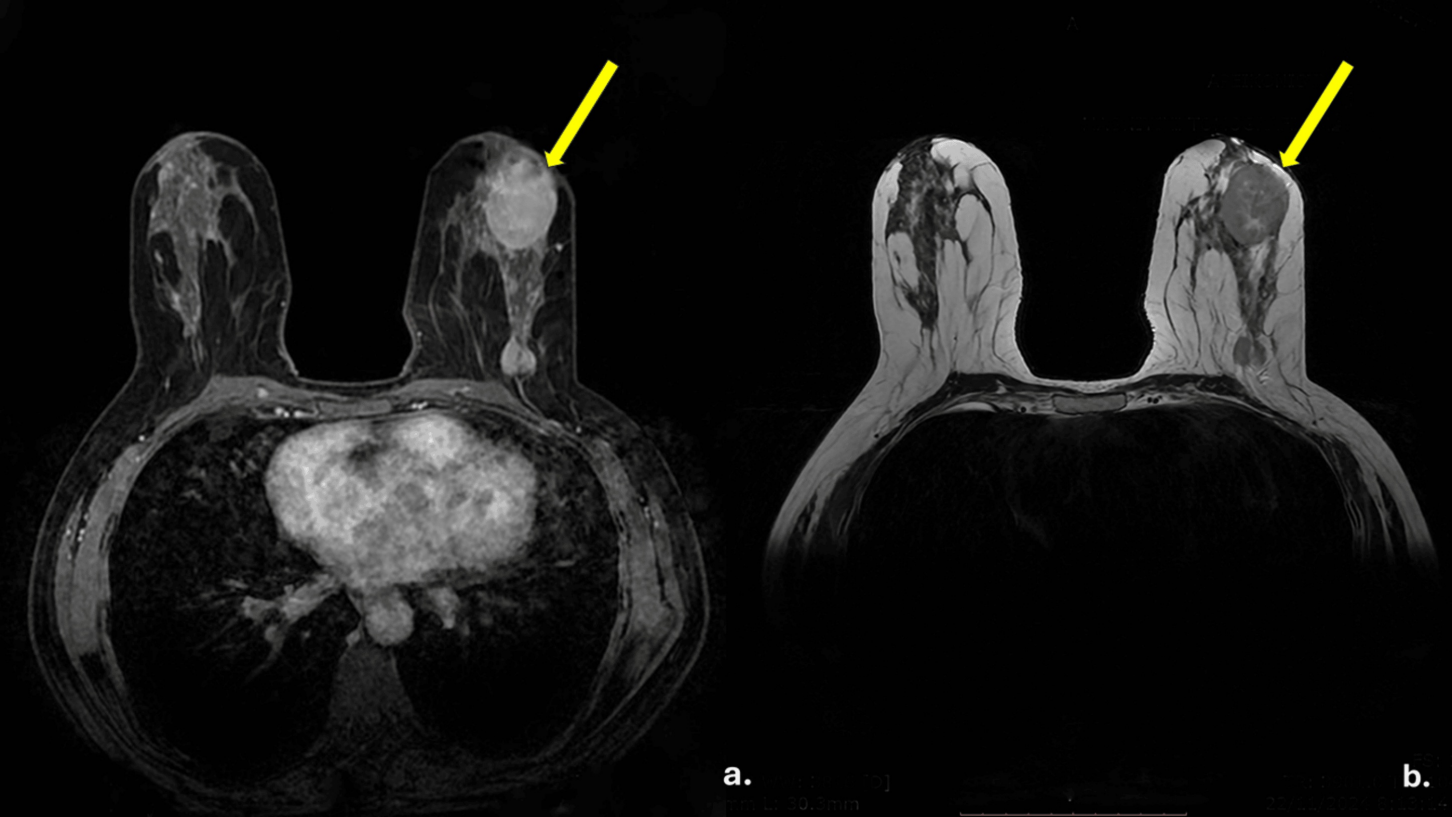
Bilateral breast MRI images The images demonstrate a large, irregular lesion (measuring 3,9 x 3,1cm) in the UOQ of the left breast (yellow arrows). (a) Axial T1-weighted post-contrast fat-suppressed image (3D GRE) shows intense enhancement of the dominant lesion (b) Axial T2-weighted fat-suppressed (STIR) image shows the same lesion as hyperintense. These findings reflect features of the BI-RADS IVa classification. UOQ: Upper outer quadrant, MRI: Magnetic resonance imaging.

MRI confirmed multifocal nodular lesions in the left breast, the largest measuring 3.9 × 3.1 cm, as seen in Figure 2, corresponding to the clinically palpable mass previously detected on ultrasound, which measured 5.98 × 2.81 cm. The slight difference in measurements was attributed to differences in imaging modalities and lesion morphology. Multiple smaller nodular lesions (ranging from 10 to 20 mm) were also identified in the left breast, consistent with multifocal disease. Lesions exhibited increased signal intensity on DWI, low ADC values (700-850 mm²/sec), and type II and III kinetic curves on dynamic contrast-enhanced sequences. Findings were classified as BI-RADS 4a, warranting further diagnostic evaluation.

**Figure 3:**
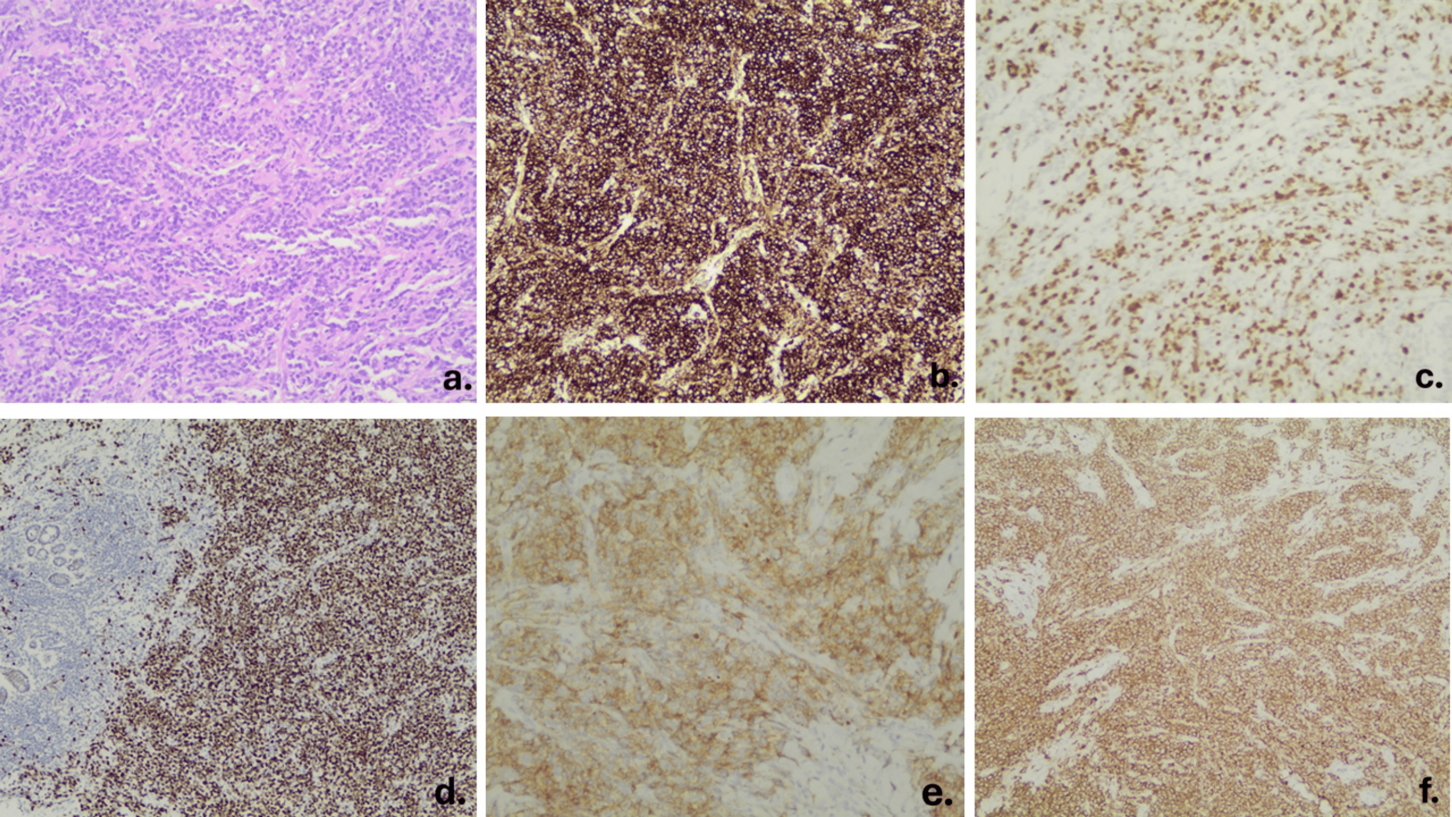
Histopathological and IHC findings from the ultrasound-guided core needle biopsy of the breast mass. (a) H&E stain (X200): diffuse infiltration by atypical lymphoid cells. (b) LCA (X100) confirms lymphoid origin. (c) Ki-67 (X200): high proliferative index (~70%). (d) BCL6 (X100) and (e) CD10 (X200): GCB phenotype. (f) CD20 (X100) confirms B-cell lineage. IHC: Immunohistochemical findings, LCA: Leukocyte common antigen.

An ultrasound-guided core needle biopsy of the primary mass confirmed diffuse large B-cell lymphoma (DLBCL) of germinal center B-cell (GCB) phenotype. Histological analysis (Figure 3) revealed a diffuse neoplastic lymphoid population composed of medium and large cells. The cells were positive for LCA and negative for CKAE1/AE3. Remnants of benign terminal duct lobular units were preserved within the neoplasm, staining positive for CKAE1/AE3, confirming that the lymphoma infiltrated the breast parenchyma, supporting the diagnosis of PBL as the primary site of lymphoma. The neoplastic cells were positive for CD20, CD10, BCL2, and BCL6, with high proliferative activity (Ki67 ~70%), and negative for CD5, CD3, CD30, and cyclin D1. The patient received eight cycles of R-CHOP chemotherapy. After four cycles, mid-treatment PET-CT, as seen in Figure 1d, demonstrated complete metabolic response, with no residual hypermetabolic lesions detected.

## Discussion

Epidemiology and pathophysiology

PBL is a rare extranodal lymphoma, accounting for 0.04%-0.5% of all breast malignancies, 1% of non-Hodgkin lymphomas (NHL), and less than 3% of extranodal lymphomas [1-3]. It originates within the breast tissue itself, without prior lymphoma or systemic involvement [1,3]. PBL occurs predominantly in women in their fifth to sixth decades of life [1], although rare cases in men have been reported [4]. Primary breast lymphoma (PBL) includes a spectrum of lymphomas. The most frequently encountered histological subtype of PBL is the DLBCL, accounting for 56-84% of cases. Other relatively common types include marginal zone lymphoma (MZL) (9-28%) and follicular lymphoma (10-19%). Burkitt lymphoma is rare, seen in less than 1% of cases. Less common subtypes, each comprising under 1% of PBLs, include anaplastic large cell lymphoma, peripheral T-cell lymphoma, small lymphocytic lymphoma, lymphoplasmacytic lymphoma, mantle cell lymphoma, and Hodgkin lymphoma [1].

Given the absence of organized lymphoid structures within the breast, its involvement is rare. Gene expression profiling using cDNA microarrays has defined molecular subgroups in DLBCL, initially distinguishing germinal center B-cell-like (GCB) and activated B-cell-like (ABC) subtypes, with a third unclassified (type 3) group reflecting additional heterogeneity. In routine practice, immunohistochemical markers including CD10, BCL6, and MUM1 are commonly used to subclassify DLBCL into GCB and non-GCB categories [2]. Although most PBL-DLBCL cases exhibit the non-GCB phenotype (reported in 60-90% of cases) [3], GCB-DLBCL-arising from germinal center B cells-can rarely occur, as seen in this case, highlighting an uncommon histologic variant of PBL.

The pathophysiology and etiology of PBL-DLBCL remain incompletely understood. However, several risk factors have been proposed that may contribute to the aberrant recruitment and proliferation of lymphoid cells within breast tissue. Hormonal changes associated with pregnancy, lactation, and menopause, as well as chronic antigenic stimulation from inflammatory or autoimmune conditions, are thought to create a microenvironment conducive to lymphoid proliferation and subsequent malignant transformation [5].

Clinical presentation and diagnostic challenges

Clinical Presentation

PBL typically manifests as a rapidly enlarging, single mobile palpable mass [4], often located in the superior quadrant [6], with or without ipsilateral axillary lymphadenopathy closely resembling the presentation of breast carcinoma [3]. Unilateral involvement is most common, although bilateral cases are reported in approximately 10-11% of patients [6]. The right breast is more frequently affected [7]. Unlike systemic lymphomas, classic B symptoms (fever, weight loss, night sweats) are rare at presentation and usually occur only in advanced stages [3]. Importantly, PBL is clinically indistinguishable from breast carcinoma as both present with a painless breast mass [1]. However, compared to carcinomas, lymphomas are often larger at diagnosis and are less frequently associated with skin retraction, nipple discharge, erythema, or peau d’orange [1,3]. In the present case, the patient’s painless, rapidly growing mass without skin or nipple changes was consistent with typical PBL features.

Mammography

On mammography, PBL typically presents as a large, well-circumscribed or ill-defined mass without microcalcifications or spiculations, often accompanied by axillary lymphadenopathy [8]. Sabaté et al. report that PBL lesions are typically larger (4-5 cm) than breast carcinomas (2-3 cm) and are less likely to exhibit architectural distortion [9]. In dense breasts (e.g., ACR Type C, as in this case), mammographic sensitivity is further reduced [8]. Here, the lesion was misclassified as BI-RADS 3 ("probably benign"), highlighting the limitations of routine imaging in detecting PBL.

Ultrasound and MRI

Ultrasound and MRI characteristics of PBL are similarly nonspecific and neither can definitively distinguish PBL from other breast malignancies, reinforcing the need for tissue diagnosis [1]. No radiologic features are definitive for diagnosis, making biopsy essential [3,9].

*Biopsy, Immunohistochemical* ​​​​​​ (*IHC), and Molecular Markers*

Histopathological and immunophenotypic analyses are essential for confirming the diagnosis of PBL. While excisional biopsy remains the gold standard, core needle biopsy under ultrasound guidance, as performed in this case, is a valid and commonly used alternative [1]. Histologically, PB-DLBCL is characterized by diffuse infiltration of large neoplastic B cells with round to oval or lobulated nuclei and often prominent nucleoli. The proliferation index of PB-DLBCL, measured by Ki-67, is typically high, often exceeding 80% [5].

PET-CT

PET-CT is crucial for staging, response evaluation, and follow-up [9]. In this case, PET-CT confirmed localized disease to the left breast and absence of systemic spread, supporting the diagnosis of PBL. It also ruled out metabolic activity in the pulmonary nodules and other extranodal sites.

Management and prognosis

Early and accurate diagnosis of PBL is essential due to its distinct management, which differs significantly from breast carcinoma [10]. Systemic chemo-immunotherapy, particularly R-CHOP, followed by radiation therapy, is the standard of care for PB-DLBCL, significantly improving local and distant disease control [1,2]. While R-CHOP is routinely used, the benefit of adding rituximab remains variable across studies [1]. Radiation therapy complements systemic treatment in PBL by reducing local recurrence and potentially enhancing progression-free and overall survival [2,10]. Its use should be tailored based on tumor size, response to systemic therapy, potential radiation-related morbidity, and salvage options. When systemic therapy achieves good disease control, targeted radiation may suffice to address residual microscopic disease. In non-curative settings, radiation can also provide effective palliation [10]. Central nervous system (CNS) involvement is rare, but individualized CNS prophylaxis may be considered [2]. Unlike breast cancer, surgery has a limited and often unfavorable role in PBL, with procedures like mastectomy linked to increased all-cause and disease-specific mortality [1]. Therefore, surgery is generally avoided beyond diagnostic excision and is not part of the standard treatment for PB-DLBCL [1,2,9]. If surgery is performed due to misdiagnosis, early initiation of chemotherapy (e.g., RCHOP) followed by radiation therapy after surgical wound healing is recommended [1].

In this case, the patient received eight cycles of R-CHOP chemotherapy. A mid-treatment PET-CT after four cycles demonstrated complete metabolic response, with no residual hypermetabolic lesions. Although breast involvement was present, CNS prophylaxis was not administered due to the absence of additional CNS risk factors such as high CNS-IPI score, multiple extranodal sites, or aggressive molecular features, and following individualized risk assessment. The patient remained in complete remission, supporting the favorable outcome achieved with systemic therapy alone.

## Conclusions

This case illustrates a rare presentation of the GCB subtype of DLBCL as a PBL, an uncommon histologic subtype, as most PBLs are non-GCB. Clinically and radiologically mimicking breast carcinoma, definitive diagnosis was achieved through biopsy and immunophenotyping, underscoring the importance of tissue sampling and molecular subtyping in atypical breast lesions. The case emphasizes the utility of a multimodal imaging approach, including ultrasound, MRI, CT, and PET-CT, in confirming localized disease and excluding systemic involvement, a critical step in diagnosing true PBL. An excellent treatment response to R-CHOP alone, without surgery, supported modern management strategies that prioritize systemic therapy and targeted radiation over surgical treatment in PBL. Given the rarity of PBL, particularly the GCB subtype, this case underscores the need for heightened clinical suspicion, early histopathologic confirmation, and a multidisciplinary, individualized management strategy.
